# Deficiency of the eIF4E isoform nCBP limits the cell-to-cell movement of a plant virus encoding triple-gene-block proteins in *Arabidopsis thaliana*

**DOI:** 10.1038/srep39678

**Published:** 2017-01-06

**Authors:** Takuya Keima, Yuka Hagiwara-Komoda, Masayoshi Hashimoto, Yutaro Neriya, Hiroaki Koinuma, Nozomu Iwabuchi, Shuko Nishida, Yasuyuki Yamaji, Shigetou Namba

**Affiliations:** 1 Graduate School of Agricultural and Life Sciences, The University of Tokyo, 1-1-1 Yayoi, Bunkyo-ku, Tokyo 113-8657, Japan

## Abstract

One of the important antiviral genetic strategies used in crop breeding is recessive resistance. Two *eukaryotic translation initiation factor 4E* family genes, *eIF4E* and *eIFiso4E*, are the most common recessive resistance genes whose absence inhibits infection by plant viruses in *Potyviridae, Carmovirus*, and *Cucumovirus*. Here, we show that another *eIF4E* family gene, *nCBP*, acts as a novel recessive resistance gene in *Arabidopsis thaliana* toward plant viruses in *Alpha-* and *Betaflexiviridae*. We found that infection by *Plantago asiatica mosaic virus* (PlAMV), a potexvirus, was delayed in *ncbp* mutants of *A. thaliana*. Virus replication efficiency did not differ between an *ncbp* mutant and a wild type plant in single cells, but viral cell-to-cell movement was significantly delayed in the *ncbp* mutant. Furthermore, the accumulation of triple-gene-block protein 2 (TGB2) and TGB3, the movement proteins of potexviruses, decreased in the *ncbp* mutant. Inoculation experiments with several viruses showed that the accumulation of viruses encoding TGBs in their genomes decreased in the *ncbp* mutant. These results indicate that *nCBP* is a novel member of the *eIF4E* family recessive resistance genes whose loss impairs viral cell-to-cell movement by inhibiting the efficient accumulation of TGB2 and TGB3.

About half of the determinant loci from natural resistant crop cultivars are recessively inherited^1^. The products of these recessive resistance genes are usually regarded as essential host factors required for the propagation of the virus. Therefore, a cultivar that lacks a host factor(s) that is indispensable for viral infection should be a recessive resistant cultivar^2^,^3^.

The most common recessive resistance genes encode eukaryotic translation initiation factor 4E and eIF4G, components of eIF4F^4^, but other types of genes have also been isolated from barley and *Arabidopsis thaliana*^5^,^6^. eIF4E and its isoform eIFiso4E are canonical cap binding proteins, which function in translation initiation in plant cells^7^. eIF4E and eIFiso4E interact with eIF4G and eIFiso4G to form the eIF4F and eIFiso4F complex, respectively. eIF4F and eIFiso4F also interact with eIF3, which recruits the 40S ribosomal subunit to the 5′ untranslated region (UTR) of mRNA, to initiate translation^8^. In plants, another eIF4E isoform, the novel cap binding protein (nCBP), has been identified, which is distantly related to eIF4E and eIFiso4E^9^,^10^ (see Supplementary Fig. S1). Since nCBP interacts with the cap *in vitro* and *in vivo*^9^,^11^, it is believed to function in translation initiation, similar to eIF4E and eIFiso4E.

eIF4E and eIFiso4E are known to be one of loss-of-susceptibility determinants to many plant viruses^2^,^12^,^13^,^14^,^15^. A loss or mutation of the *eIF4E* or *eIFiso4E* genes in *Capsicum* spp., *Solanum lycopersicum*, and *A. thaliana* conferred full resistance against potyviruses^4^,^14^,^16^,^17^,^18^. Mutation in *eIF4E* in melon also resulted in an immunity type of resistance against a carmovirus, *Melon necrotic spot virus* (MNSV)^19^,^20^ and in barley against two bymoviruses, *Barley yellow mosaic virus* and *Barley mild mosaic virus*^12^. *A. thaliana* mutants of the *eIF4E* gene were also partially resistant to *Cucumber mosaic virus* (CMV, *Cucumovirus*)^21^. eIF4E was shown to be involved in the translation of viral proteins from MNSV and CMV RNAs^21^,^22^. Thus, although the roles of some recessive resistance genes have been partially elucidated, understanding of the variety of recessive resistance genes and their roles remains limited.

*Alpha-* and *Betaflexiviridae* are groups of flexuous, filamentous viruses that predominantly infect plants, and encode an RNA-dependent RNA polymerase (RdRp), a 30K-type movement protein (MP) or triple-gene-block (TGB)-type MPs, and a coat protein (CP). Some viruses in *Alpha-* and *Betaflexiviridae* encode additional proteins in their genome. The most extensively studied of these plant viruses are members of the genus *Potexvirus* in the family *Alphaflexiviridae*, which have one genomic RNA with a cap and poly(A) tail^23^,^24^,^25^. The 5′-terminal open reading frame (ORF) encoding RdRp is translated directly from genomic RNA, but the 3′-proximal ORFs encoding TGB1, TGB2, TGB3, and CP are translated from subgenomic RNAs (sgRNAs)^26^,^27^, which are generated during virus replication^28^ and possess a cap^29^,^30^ and the same 3′ ends as the genomic RNA^25^. TGB1 and CP were shown to be translated from sgRNA1 and sgRNA3, respectively, while TGB2 and TGB3 are translated from sgRNA2 of *Potato virus X* (PVX)^26^,^27^.

In this study, we found that an *A. thaliana* mutant of another member of the *eIF4E* family gene, *nCBP*, was resistant to a potexvirus, *Plantago asiatica mosaic virus* (PlAMV). Cell-to-cell movement of PlAMV was delayed in an *ncbp* mutant compared to the wild type, although viral accumulation in single cells of the mutant and the wild type did not differ. The accumulation of TGB2 and TGB3 was decreased in the *ncbp* mutant compared with the wild type.

## Results

### PlAMV propagation was delayed in *ncbp* mutants

**Figure 1:**
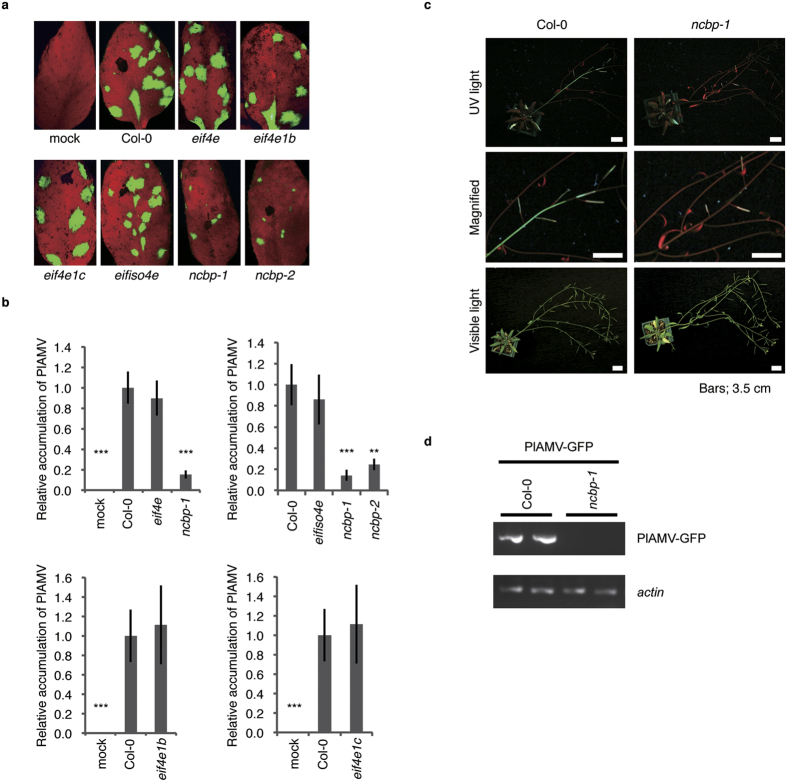
Inhibition of PlAMV propagation in *ncbp* mutants. (**a**) Green fluorescence emission of PlAMV-GFP in inoculated leaves of *A. thaliana* mutant lines of *eIF4E* family genes. Mutant lines and Col-0 were inoculated mechanically with PlAMV-GFP-infected leaf extracts and photos were taken under a fluorescence microscope at 4 days post inoculation (dpi). Representative images are shown. (**b**) PlAMV RNA accumulation in mutant lines measured by quantitative RT-PCR. Total RNA extracted from inoculated leaves in (**a**) at 4 dpi was subjected to quantitative RT-PCR using RdRp-specific primers to detect viral genomic RNA. The accumulation of PlAMV-GFP RNA normalized relative to that of *actin* mRNA is presented in each sample. The mean level of viral RNA in Col-0 was used as the standard (1.0). Error bars represent standard errors of 12 measurements from three independent experiments. Double asterisk indicates a significant difference compared with Col-0 (two-tailed Dunnett’s test, double asterisk; P < 0.01, triple asterisk; P < 0.001). (**c**) Fluorescence images of PlAMV-GFP in systemic plants. PlAMV-GFP was inoculated mechanically onto *ncbp-1* and Col-0 and photos of fluorescence (upper and middle panels) and bright-field (lower panels) images of systemic plants were taken at 21 dpi. Bars, 3.5 cm. Middle panels are magnified images of upper panels. (**d**) Detection of PlAMV-GFP by RT-PCR in upper leaves. Upper leaf samples in (**c**) at 21 dpi were analyzed by RT-PCR using CP-specific primers with the *actin* gene as an internal control. Cropped gel images are shown and full-length gel images are included in Supplementary Figure S7. Experiments were replicated three times.

*A. thaliana* has three types of eIF4E isoforms, eIF4E, eIFiso4E, and nCBP. Two homologs of eIF4E, eIF4E1b and eIF4E1c, are also encoded by *A. thaliana*^31^ (see Supplementary Fig. S1). To determine whether any of the eIF4E isoforms has a role in the infection cycle of PlAMV, *A. thaliana* mutant lines bearing a T-DNA insertion or a point mutation in an *eIF4E* family gene were mechanically inoculated with PlAMV-GFP, a GFP-expressing variant of PlAMV. The inoculated leaves were examined for fluorescence from PlAMV-GFP at 4 days post inoculation (dpi). Infection foci on the inoculated leaves of *eif4e, eif4e1b, eif4e1c*, and *eifiso4e* mutants were nearly the same size as those on the *A. thaliana* ecotype Columbia-0 (Col-0) leaves (Fig. 1a). In contrast, the foci on the inoculated leaves of two *ncbp* T-DNA insertion lines, *ncbp-1* and *ncbp-2* (see Supplementary Fig. S2), were noticeably smaller than those of Col-0 (Fig. 1a). To quantify the virus accumulation, total RNA was extracted from each of the inoculated leaves from the mutants and analyzed by quantitative RT-PCR (RT-qPCR) with PlAMV- specific primers^32^. The relative accumulation of PlAMV viral RNA in *eif4e, eif4e1b, eif4e1c*, and *eifiso4e* mutants did not differ from that in Col-0, whereas viral RNA in *ncbp-1* and *ncbp-2* mutants was drastically decreased to approximately 15% and 25% of that in Col-0, respectively (Fig. 1b).

To explore whether PlAMV systemically infects *ncbp* mutants, we mechanically inoculated *ncbp* mutants and Col-0 with PlAMV-GFP. At 3 weeks post inoculation (wpi), the GFP signal was observed in stems and upper leaves of Col-0, but no GFP signal was apparent in the upper leaves of the *ncbp-1* mutant (Fig. 1c). To quantify the accumulation of viral RNA, we extracted total RNA from non-inoculated upper leaves of the *ncbp-1* and *ncbp-2* mutants, and Col-0, and amplified viral RNA by RT-PCR. At 3 wpi, PlAMV infected the Col-0 plants systemically, while PlAMV did not propagate in upper leaves of the *ncbp-1* and *ncbp-2* mutants (see Supplementary Table S1 and Fig. 1d). At 4 wpi, 3 of 5 *ncbp-1* plants, and none of the 6 *ncbp-2* plants, harbored PlAMV RNA (see Supplementary Table S1). The accumulation of PlAMV in the upper leaves of the *ncbp-1* mutants at 4 wpi was lower than in Col-0 plants (see Supplementary Fig. S3), suggesting that PlAMV systemic infection delayed in these mutants.

**Figure 2:**
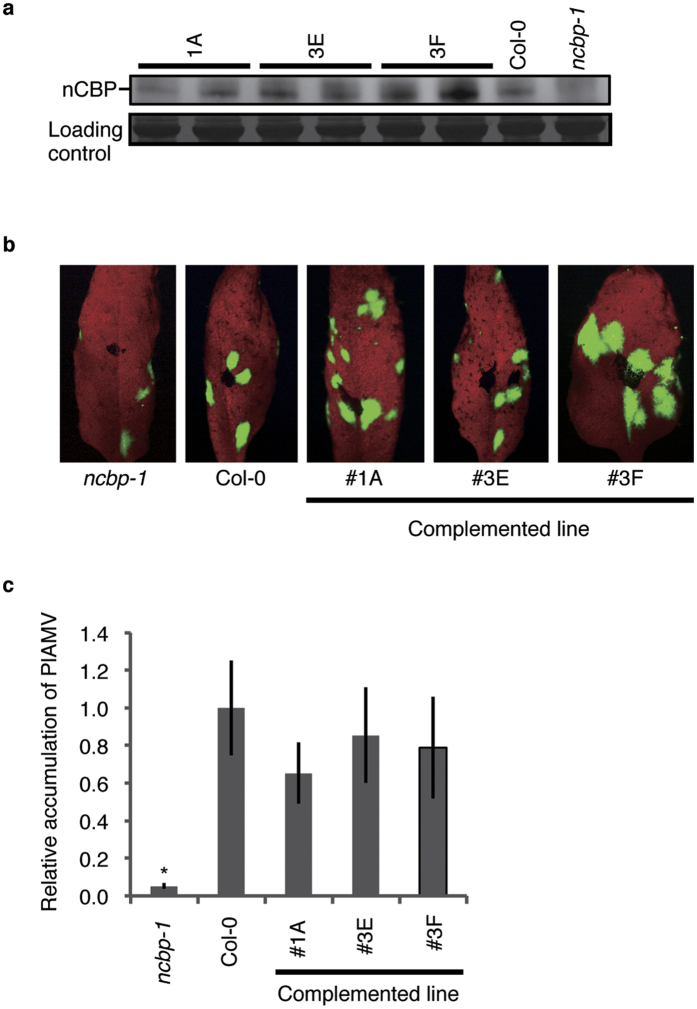
Functional validation of nCBP by transgenic complementation. (**a**) Accumulation of nCBP protein in *A. thaliana* transformants. Total protein was extracted from rosette leaves of Col-0, the *ncbp-1* mutant, and three nCBP-complemented transgenic lines, and analyzed by immunoblotting, using antisera recognizing nCBP. The cropped image is shown and the full-length blot is included in Supplementary Figure S8. Experiments were replicated twice. (**b**) Green fluorescence emission from PlAMV-GFP-inoculated leaves of *A. thaliana* transformants. Transformant lines as well as Col-0 and *ncbp-1* mutants were inoculated mechanically with PlAMV-GFP and photos were taken at 4 dpi. Representative images are shown. (**c**) PlAMV RNA accumulation in inoculated leaves of *A. thaliana* transformants. Total RNAs extracted from inoculated leaves of corresponding plants in (**b**) at 4 dpi were analyzed based on quantitative RT-PCR using RdRp-specific primers. PlAMV RNA accumulation was normalized relative to the *actin* mRNA value in each sample. The mean level of viral RNA in Col-0 was used as the standard (1.0). Error bars represent standard errors of at least 10 measurements from three independent experiments and the asterisk indicates a significant difference compared with Col-0 (two-tailed Dunnett’s test, asterisk; P < 0.05).

To confirm that the decreased accumulation of PlAMV in the *ncbp* mutants was due to the loss of nCBP, we cloned and introduced the genomic DNA sequence of *nCBP* with its possible promoter and terminator regions into the *ncbp-1* mutant. Three independent transgenic lines (#1A, #3E, and #3F) were obtained and analyzed for their expression of nCBP. While we failed to detect the nCBP protein in the *ncbp-1* mutant, three transgenic lines expressed it (Fig. 2a). When those transgenic lines were then inoculated mechanically with PlAMV-GFP, all of the nCBP-complemented lines had GFP foci similar in size to those of Col-0 at 4 dpi (Fig. 2b). The RT-qPCR analysis confirmed that the accumulation level of viral RNA in the inoculated leaves did not drastically differ between the transgenic lines and Col-0 (Fig. 2c). These results suggest that nCBP is essential for the efficient accumulation of PlAMV in inoculated leaves. We also confirmed that PlAMV systemically infects these complemented lines at 3 wpi (see Supplementary Table S1).

### Cell-to-cell movement of PlAMV is delayed in *ncbp* mutant

**Figure 3:**
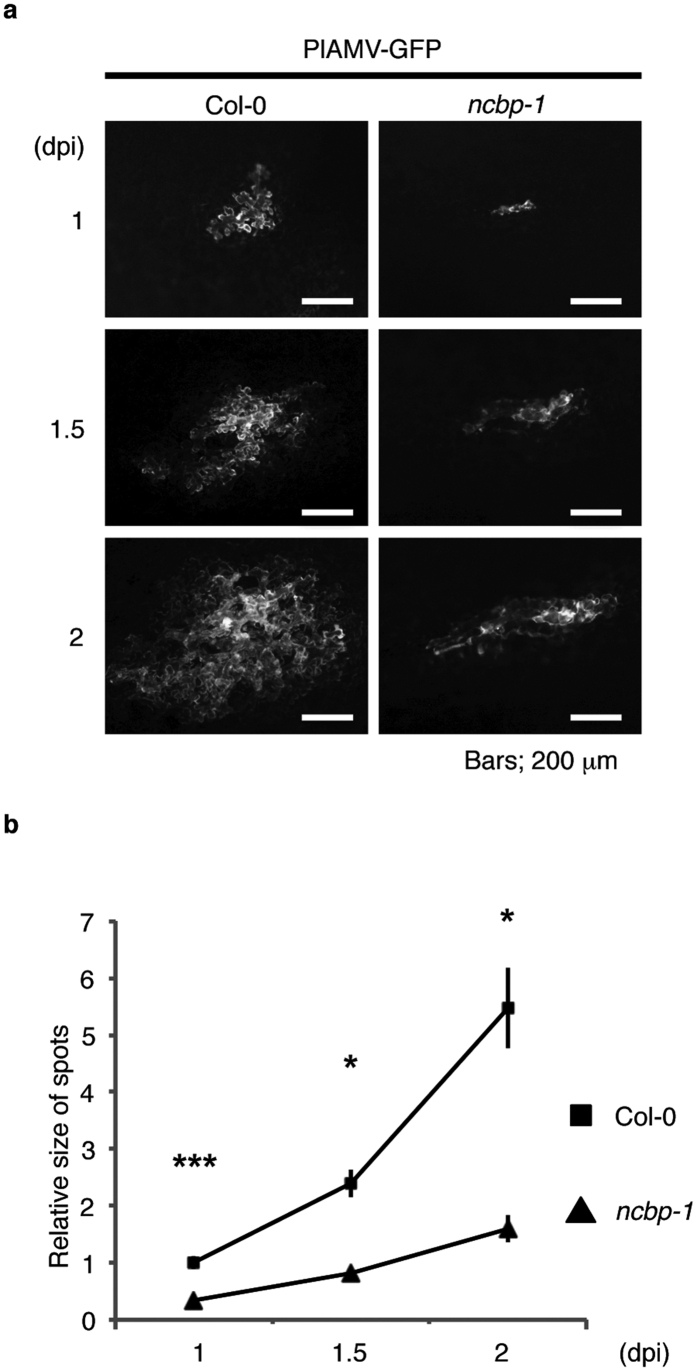
Inhibition of cell-to-cell movement of PlAMV in the *ncbp* mutant. (**a**) Images of the spread of PlAMV-GFP in epidermal cells. Rosette leaves of Col-0 and the *ncbp-1* mutant were inoculated with PlAMV-GFP using particle bombardment. Fluorescence images were obtained at 1, 1.5, and 2 dpi. Representative images are shown. Bars, 200 μm. (**b**) Quantification of the size of the fluorescent foci in inoculated leaves. Fluorescence images of more than five foci in (**a**) were processed using ImageJ software v1.40 (NIH) to measure the size of viral infection foci. The sizes are normalized to Col-0 at 1 dpi. Error bars represent standard errors of at least six measurements. Asterisk indicates a significant difference compared with Col-0 (two-tailed Student’s t-test, asterisk; P < 0.05, triple asterisk; P < 0.001). Experiments were replicated three times.

To analyze the efficiency of cell-to-cell movement of PlAMV, we monitored the sizes of PlAMV-GFP infection foci in inoculated leaves of Col-0 and the *ncbp-1* mutant. We often observed the fusion of multiple infection foci during the mechanical inoculation assay, which limits our statistical evaluation of the infection foci size (Figs 1a and 2b). Therefore, we utilized a particle delivery system to introduce the PlAMV-GFP cDNA into a single cell. The bombarded leaves were analyzed at 1, 1.5, and 2 days post bombardment. The PlAMV-GFP in the bombarded leaves of Col-0 spread more rapidly than in those of the *ncbp-1* mutant (Fig. 3a). To quantitatively analyze viral cell-to-cell movement, we measured the area expressing GFP signals. We found that the GFP-expressing area in the *ncbp-1* mutant was drastically smaller than that in Col-0 (Fig. 3b). These results suggest that the cell-to-cell movement of PlAMV was delayed in the *ncbp-1* mutant compared to Col-0.

### Replication efficiency of PlAMV is not compromised in *ncbp-1* mutant at a single-cell level

**Figure 4:**
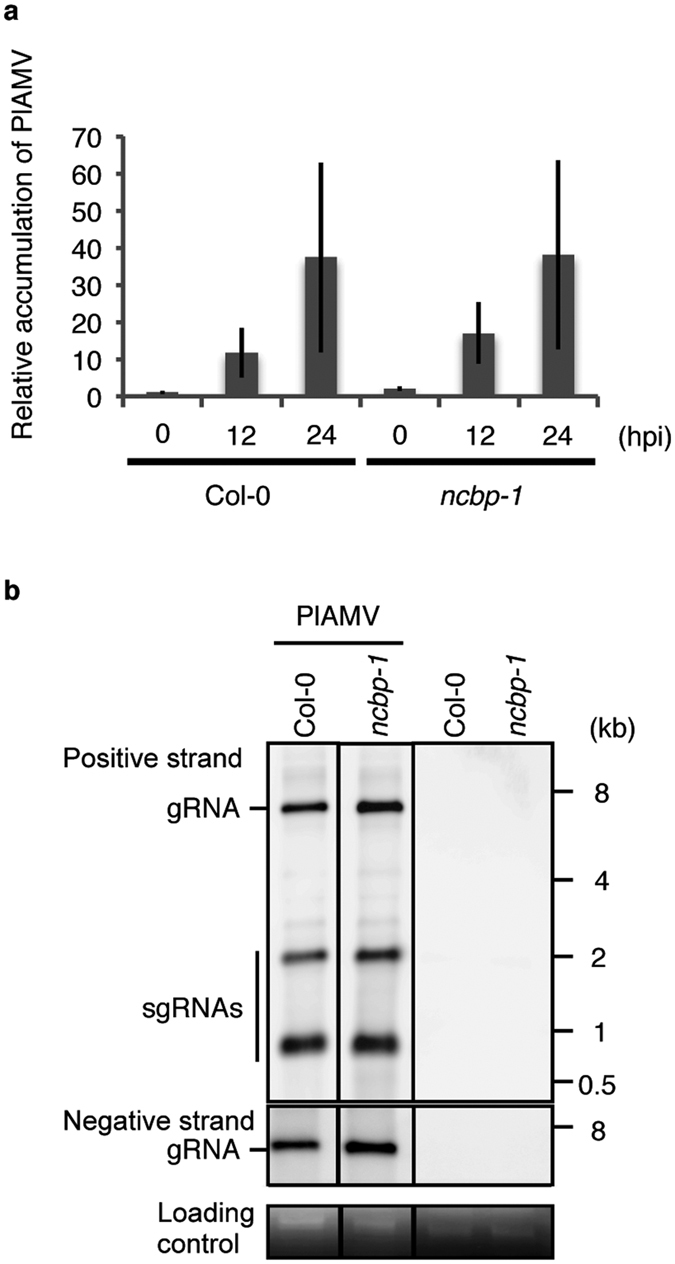
Loss of nCBP does not interfere with PlAMV RNA accumulation at the single-cell level. (**a**) Relative accumulation of PlAMV RNA at the single cell level measured by quantitative RT-PCR. Protoplasts prepared from the *ncbp-1* mutant and Col-0 were transfected with a PlAMV-containing plasmid together with a *GFP*-expressing plasmid. Extracted total RNAs from protoplast samples at three different time points were analyzed using RT-qPCR. The level of PlAMV RNA accumulation was normalized to that of *GFP* mRNA in each sample. The mean level of viral RNA in Col-0 immediately after inoculation (0 hpi) was used as the standard (1.0). Error bars represent standard errors of three independent experiments. (**b**) Detection of viral RNAs by northern blot analysis. Protoplasts from *ncbp-1* and Col-0 were inoculated with PlAMV. Total RNA (1 μg) extracted from protoplast samples at 3 dpi was subjected to northern blot analysis using specific probes. Cropped images are shown and the full-length blots are presented in Supplementary Figure S9. Uninoculated controls are indicated. Bands of ribosomal RNA are shown as loading controls. Experiments were replicated three times.

We next investigated whether the delay of viral cell-to-cell movement in the *ncbp* mutants was caused by a decreased replication efficiency of the virus. We isolated mesophyll protoplasts from the *ncbp-1* mutant and Col-0 and transfected them with infectious PlAMV cDNA. Total RNA was extracted from the cells at 0, 12, and 24 hours post inoculation (hpi), and the amount of viral RNA was analyzed by RT-qPCR using the cotransfected *GFP* gene as an internal control. RT-qPCR analysis revealed that the accumulation level of viral RNA in the *ncbp-1* mutant protoplasts was similar to that in Col-0 protoplasts (Fig. 4a). We further performed northern blot analysis to detect viral genomic RNA, sgRNAs, and negative-strand viral RNA that serves as a template for synthesis of the RNA genome. We found that viral genomic RNA, sgRNAs and negative strand RNA accumulated to a similar degree in both the *ncbp-1* and Col-0 protoplasts (Fig. 4b, top and middle panels, Supplementary Fig. S4). These results show that nCBP is not an essential factor for PlAMV genomic replication, suggesting that nCBP is involved in viral cell-to-cell movement.

### Accumulation of TGB2 and TGB3 is decreased in *ncbp* mutant

**Figure 5:**
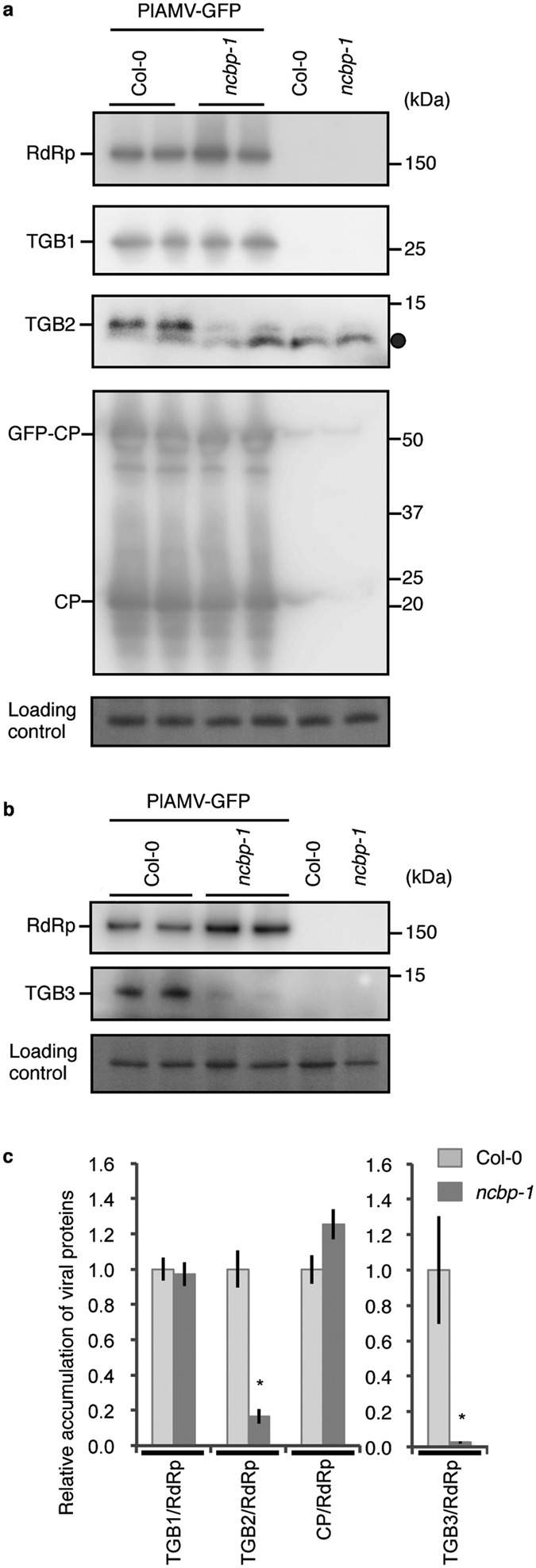
Decreased accumulation of TGB2 and TGB3 in the *ncbp* mutant. (**a**) Accumulation of PlAMV proteins in the *ncbp-1* mutant and Col-0. *ncbp-1* mutant and Col-0 plants were agro-inoculated with PlAMV-GFP. Total protein was extracted from inoculated leaves at 4 dpi and subjected to immunoblot analysis using antibodies against TGB1, CP, and RdRp, and antisera recognizing TGB2. Two experimental replicates for PlAMV-agroinfiltrated leaves along with uninoculated controls are indicated. A closed circle indicates nonspecific bands that could also be detected in negative control lanes. Cropped images are shown and the full-length blots are presented in Supplementary Figure S8. Experiments were repeated three times. (**b**) Accumulation of RdRp and TGB3 proteins in the P30 fraction from the *ncbp-1* mutant and Col-0. Total protein was extracted from the agro-inoculated leaves and subjected to ultracentrifugation. The resulting P30 fraction was analyzed by immunoblotting, using an antibody recognizing RdRp and TGB3. Cropped images are shown and the full-length blots are presented in Supplementary Figure S8. The experiments were replicated twice. (**c**) Relative accumulation of TGB1, TGB2, TGB3, and CP in virus-infected *ncbp-1* mutant and Col-0. The intensities of the bands for TGB1, TGB2, TGB3, and CP were measured and normalized to that for RdRp. Accumulation level in Col-0 was used as the standard (1.0). Error bars represent standard errors of five measurements. The asterisk indicates a significant difference compared with Col-0 (two-tailed Student’s t-test, asterisk; P < 0.05).

Since potexviruses need TGB1, TGB2, TGB3 and CP for their cell-to-cell movement, we analyzed the accumulation of these viral proteins in PlAMV-transfected protoplasts of the *ncbp-1* mutant and Col-0. Immunoblotting of the protoplast samples harvested at 3 dpi revealed that the accumulation of RdRp did not differ between the *ncbp-1* and Col-0 protoplasts (see Supplementary Fig. S5). However, we could not clearly detect TGB2 and TGB3 proteins in protoplast samples (see Supplementary Fig. S5), possibly because of their low accumulation levels. Therefore, we used an Agrobacterium-mediated transient expression (agroinfiltration) assay in which infiltrated regions of plant leaves were infected uniformly with virus. Leaves of the *ncbp-1* mutant and Col-0 were agroinfiltrated with *Agrobacterium* carrying infectious PlAMV-GFP cDNA. The leaves were harvested at 4 dpi and the accumulation of viral proteins was analyzed by immunoblotting (Fig. 5a). Since the band for TGB3 exactly overlapped with a nonspecific band, we separated soluble and insoluble fractions (S30 and P30 fraction, respectively) by ultracentrifugation to exclude the nonspecific band based on the fact that the TGB3 protein is membrane-associated^33^. The nonspecific band was detected only in the S30 fraction, enabling us to evaluate the accumulation of TGB3 in the P30 fraction (see Supplementary Fig. S6 and Fig. 5b). Although the accumulation of RdRp, TGB1, GFP-CP and CP was similar between Col-0 and the *ncbp-1* mutant plants, that of TGB2 and TGB3 drastically decreased in the *ncbp-1* mutant compared to that in Col-0 (Fig. 5a and b). The intensities of protein bands in each leaf sample were quantified (Fig. 5c). We evaluated the ratio of TGB1, TGB2, TGB3, and CP accumulation relative to that of RdRp, since the amount of viral RNA and RdRp was almost the same between the *ncbp-1* mutant and Col-0 protoplasts (see Supplementary Fig. S5 and Fig. 4). Band quantification confirmed that the amounts of TGB2 and TGB3 drastically decreased in *ncbp-1* leaves compared to Col-0 leaves, while the relative amount of TGB1 and CP in the *ncbp-1* leaves did not differ (Fig. 5c).

### nCBP is required for efficient propagation of viruses in *Alphaflexiviridae* and *Betaflexiviridae*

**Figure 6:**
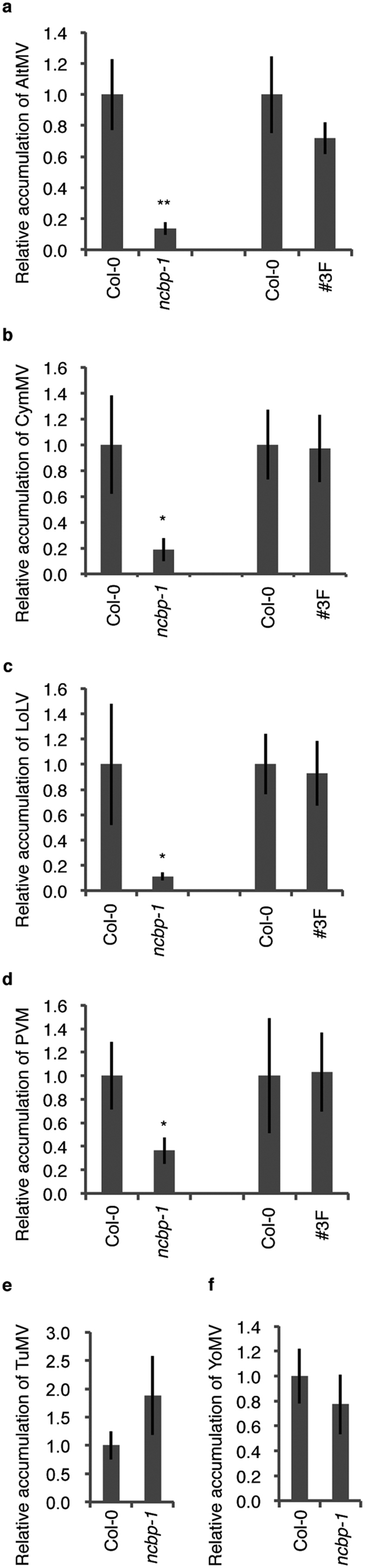
nCBP is required for the efficient accumulation of viruses in *Alphaflexiviridae* and *Betaflexiviridae*. The *ncbp-1* mutant, Col-0, and the nCBP-complemented line (3 F) were inoculated mechanically with AltMV (**a**), CymMV (**b**), LoLV (**c**), PVM (**d**), TuMV (**e**), and YoMV (**f**), and total RNA was extracted from inoculated leaves at 4 dpi. Virus accumulation was analyzed by quantitative RT-PCR using virus-specific primers. The accumulation of viral RNA was normalized relative to *actin* mRNA in each sample. The mean level of viral RNA in Col-0 was used as the standard (1.0). Error bars represent standard errors of 12 measurements from three independent experiments and asterisk and double asterisks indicate significant differences compared with Col-0 (one-tailed Student’s t-test, asterisk; P < 0.05, double asterisk; P < 0.01).

To determine which viruses require nCBP for their efficient accumulation, we mechanically inoculated the *ncbp-1* mutant and Col-0 with viruses from various families and tested their accumulation. In *Alphaflexiviridae*, we analyzed two potexviruses (*Alternanthera mosaic virus* [AltMV] and *Cymbidium mosaic virus* [CymMV]) and a lolavirus (*Lolium latent virus* [LoLV]). Furthermore, a carlavirus (*Potato virus M* [PVM]) in *Betaflexiviridae*, a potyvirus (*Turnip mosaic virus* [TuMV]) in *Potyviridae*, and a tobamovirus (*Youcai mosaic virus* [YoMV]) in *Virgaviridae* were also investigated. The inoculated leaves were harvested at 4 dpi, and total RNA extracted from each leaf was analyzed using RT-qPCR with primers specific to each virus. Accumulation of AltMV, CymMV, LoLV, and PVM in the *ncbp-1* mutant decreased drastically to 14%, 18%, 11%, and 42% of that in Col-0, respectively (Fig. 6a–d), suggesting that nCBP is required for the efficient accumulation of viruses belonging to the *Alphaflexiviridae* and *Betaflexiviridae* families. We confirmed that the accumulation of AltMV, CymMV, LoLV, and PVM were recovered in the nCBP-complemented line #3F (Fig. 6a–d). In contrast, TuMV and YoMV propagated in the *ncbp-1* mutant to a similar level as in Col-0 (Fig. 6e,f).

## Discussion

We identified *nCBP* as a novel recessive resistance gene against plant viruses in *Alphaflexiviridae* and *Betaflexiviridae*. This identification revealed that all members of the *eIF4E* family (*eIF4E, eIFiso4E*, and *nCBP*) can act as recessive resistance genes.

Recessive resistance exhibited by eIF4E deficiency is thought to be caused by the specific use of *eIF4E* family gene products, eIF4E or eIFiso4E, by plant viruses^2^. Generally, plants encode three eIF4E isoforms, namely, eIF4E, eIFiso4E, and nCBP^10^. The lack of eIF4E or eIFiso4E does not influence the viability of plants^16^,^31^,^34^,^35^, presumably due to their redundancy during translation initiation. However, the lack of eIF4E or eIFiso4E does decrease the infectivity of plant viruses; therefore, such viruses are thought to use specific *eIF4E* family gene products during their infection^2^. The specific use of *eIF4E* family gene products may be correlated with the unique translation initiation strategies of plant viruses. In fact, a large number of plant viruses do not possess the cap and/or poly(A) structures in their genomic RNA^36^. Because both of these structures play a critical role in translation initiation^37^, these viruses developed unique translation initiation strategies, e.g., recruiting specific eIFs directly to viral RNAs using their own *cis*-acting RNA elements^36^. Examples include the 3′ cap-independent translation element (3′ CITE) located within the 3′ UTR of viruses in Tombusviridae, *Umbravirus*, and *Luteovirus*. Each virus recruits specific eIFs to the 3′ CITE to facilitate the translation of viral proteins^38^. Therefore, eIF4E-mediated recessive resistance is effective against plant viruses lacking cap and/or poly(A) structures^4^,^19^,^36^. In this study, we showed that the infection of plant viruses with cap and poly(A) structures was inhibited in *ncbp* mutant plants. Our results showed that *eIF4E* family genes can serve as recessive resistance genes against viruses with cap and poly(A) structures.

In this study, we showed that cell-to-cell movement of PlAMV was inhibited in the *ncbp* mutant (Fig. 3); thus, we further analyzed the role of nCBP during PlAMV infection. Protoplast transfection assays revealed no significant difference between the *ncbp-1* and Col-0 cells in the accumulation of PlAMV genomic RNA (Fig. 4). This result indicates that nCBP is not required for viral replication at the single-cell level, including translation of viral RdRp and its viral genomic RNA synthesis. In agreement with this result, the level of RdRp in PlAMV-agroinfiltrated leaves of the *ncbp* mutant was similar to that of Col-0 (Fig. 5a). However, the accumulation of TGB2 and TGB3 drastically decreased in PlAMV-infiltrated *ncbp* mutant leaves (Fig. 5a and b), indicating that decreased accumulation of these proteins might cause the inefficient cell-to-cell movement of the virus. It still remains unclear why the accumulation of TGB2 and TGB3 decreased in the *ncbp* mutant. Considering that nCBP is a member of eIF4E isoforms, it is attractive to think that the translation of TGB2 and TGB3 from sgRNA2 may be specifically inactivated in the *ncbp* mutant, becuase sgRNA2 was shown to function as a template for translation of TGB2 and TGB3 in the case of PVX^26^,^27^. Otherwise, the stability of TGB2 and TGB3 could be reduced in the mutant. However, since sgRNA2 of PlAMV was below the detectable level in our northern blot analysis (Fig. 4b), it remains also possibile that the synthesis and/or stability of sgRNA2 might be affected during PlAMV infection in the *ncbp* mutant.

In this study, we showed that nCBP-mediated recessive resistance limits viral cell-to-cell movement (Fig. 3). During viral cell-to-cell movement, three potexviral movement proteins, TGB1, TGB2, and TGB3, function in a concerted manner. TGB2 and TGB3 induce formation of ER-derived TGB2/3 vesicles, which are subsequently directed to plasmodesmata (PD)^39^,^40^. TGB1 accumulates at the PD only when TGB2 and TGB3 are expressed^33^,^40^. TGB1 and TGB2 can increase the PD size exclusion limit^41^,^42^. In addition to TGBs, potexviral CP is considered an essential factor to move viral RNA between cells^43^,^44^. TGB2/3 vesicles, TGB1, CP, and viral RNA form a layered complex at the PD opening with ER membranes, possibly to promote efficient movement of the viral ribonucleoproteins^40^. The lack of any one of these movement-associated proteins should disable cell-to-cell movement of potexviruses. Therefore, the delay of cell-to-cell movement of PlAMV in the *ncbp* mutant can be explained by the inefficient accumulation of TGB2 and TGB3. Involvement of the eIF4E protein family in translation of movement proteins was reported in CMV^21^. In *A. thaliana cum-1* mutant, which possesses a nonsense mutation in the eIF4E coding sequence, inefficient translation of the CMV 3a movement protein resulted in the inhibition of cell-to-cell movement of the virus^21^. The observation that two unrelated viruses, CMV and PlAMV, utilize specific eIFs for the accumulation of MP supports the importance of controlling viral MP accumulation.

To explore the universal role of nCBP in the plant-virus interaction, we inoculated *ncbp* mutants with viruses from various genera and examined their accumulation levels. We showed that viruses in the genera *Potexvirus, Lolavirus*, and *Carlavirus* require nCBP for their accumulation, whereas viruses in the genera *Potyvirus* and *Tobamovirus* do not (Fig. 6). One noticeable characteristic common among potexvirus, lolavirus, and carlavirus is that they encode TGB-type MPs. Considering that nCBP was required for the accumulation of TGB2 and TGB3 of PlAMV (Fig. 5), nCBP may also facilitate the accumulation of TGB2 and TGB3 from lolavirus and carlavirus to promote their movement. Since there are some genera of plant viruses other than *Alphaflexiviridae* and *Betaflexiviridae*, such as *Hordeivirus, Pomovirus, Pecluvirus*, and *Benyvirus*, that encode TGB proteins^26^, it would be interesting to explore whether these viruses require nCBP for their infection. It would also be interesting to explore whether viruses not encoding TGBs in *Alphaflexiviridae* and *Betaflexiviridae* are influenced by the nCBP mutation. Moreover, it is possible that the *ncbp* mutation is a determinant of the actual recessive resistance of crop cultivars against TGB-encoding viruses, in which the responsible genes for the resistance remain unknown. In addition, the artificial introduction of mutations in the *nCBP* gene may be a novel and robust strategy to provide crops with a virus-resistant trait, similar to eIF4E or eIFiso4E.

## Methods

### Plant materials and growth conditions

Seeds of *A. thaliana* mutants, *eif4e (cum1-1*^21^), *eif4e1b* (SALK_101805C), *eif4e1c* (SALK_053503C), *ncbp-1* (SALK_131503C), and *ncbp-2* (SALK_146604), were purchased from the *Arabidopsis* Biological Resource Center (ABRC; Ohio State University, Columbus, OH). The dSpm insertion line, *eifiso4e*^35^ was kindly provided by Dr. Karen S. Browning (The University of Texas at Austin). *A. thaliana* was maintained in the growth chamber with 16-h-light/8-h-dark conditions at 22 °C throughout the assays.

### Antibodies

Anti-TGB2 and TGB3 antisera were raised in rabbits using purified peptides (TGB2, GDNLHALPHGGRY; TGB3, KQTLHHGTQPSTDL) as antigen (eurofinsgenomics, Tokyo, Japan). The nCBP protein was expressed in *Escherichia coli*, using a pET30a vector, and the purified recombinant protein was used as an antigen. Anti-TGB1, CP and RdRp antibodies were prepared as described previously^32^,^43^,^45^.

### Plasmid construction

An infectious cDNA clone and a GFP-expressing vector of a PlAMV isolate^46^ were constructed as described previously^47^,^48^. For the complementation assay, the *nCBP* gene with putative promoter and terminator sequences was PCR-amplified with primers Sl-At5g18110-up1374F and Nt-At5g18110-down1011R using total DNA extracted from Col-0 as template (for primer sequences, see Supplementary Table S2). The amplified product was digested with *Sal*I and *Not*I restriction enzymes and inserted between *Sal*I and *Not*I sites of pENTA^49^. To produce pFAST01-nCBPg, the region between attL1 and attL2 containing the nCBP expression cassette was cloned into a binary plasmid vector pFAST01 (Inplanta Innovations Inc., Kanagawa, Japan) using Gateway LR Clonase II enzyme mix (Thermo Fisher Scientific, Massachusetts, USA).

To produce RNA probes for the detection of positive- and negative-stranded viral RNA, the PCR-amplified fragment of the 3′ terminal region of a PlAMV isolate^46^ (nucleotides from 5,101 to 6,102) was cloned into pCR-Blunt II-TOPO vector (Thermo Fisher Scientific), generating pCR-Pr-1 (in antisense orientation behind the T7 promoter to produce the negative-stranded RNA detection probe) and pCR-Pr-2 (in sense orientation to produce the positive-stranded RNA detection probe).

### Virus isolate and inoculation

Mechanical inoculation with an extract of PlAMV-GFP-infected *N. benthamiana* plants and agroinoculation of PlAMV-GFP was performed as described previously^50^. CymMV (accession number, LC125633), AltMV^51^, LoLV^52^, YoMV (MAFF number 104033; National Institute of Agribiological Sciences GenBank), PVM (MAFF number 307027), TuMV^53^, and CMV^54^ were also used for mechanical inoculation. Rosette leaves of three-week-old *A. thaliana* were inoculated with extracts of the upper leaves of *N. benthamiana* or *A. thaliana* plants, which were inoculated with each virus and infected systemically.

### Plant transformation

*Agrobacterium tumefaciens* strain EHA105 carrying pFAST01-nCBPg was used for transformation of *A. thaliana* by the floral-dip method, as described previously^55^. T1 seeds of transgenic plants were selected by GFP fluorescence expressed from the seed-specific OLE1 promoter encoded by the pFAST01-nCBPg.

### Alignment and phylogenetic analysis of nCBP proteins

Sequences of *eIF4E* family genes, excluding *AteIF4E1b* and *AteIF4E1c*, were obtained from EST databases of the National Center for Biotechnology Information (NCBI, http://www.ncbi.nlm.nih.gov) and Sol Genomics Network (http://solgenomics.net). Predicted cDNA sequences of *AteIF4E1b* and *AteIF4E1c* were obtained from The *Arabidopsis* Information Resource (TAIR, https://www.arabidopsis.org). Amino acid alignments of the core region^10^ (from His-37 to His-200 in *Homo sapiens* eIF4E) were preformed using ClustalW software. Phylogenetic trees were constructed from nucleotide alignments of the core region using the neighbor-joining and boot-strapping algorithms within the Mega 6.0 software.

### Quantitative RT-PCR and RT-PCR

Total RNA was extracted from inoculated leaves or transfected protoplasts using Sepasol-RNA I solution (nacalai tesque, Kyoto, Japan). Total RNA was subjected to DNase treatment (Roche, Basel, Switzerland) followed by reverse transcription using the High Capacity cDNA Reverse Transcription Kit (Thermo Fisher Scientific). For RT-qPCR, viral RNA was amplified using the Thermal Cycler Dice Real Time System (TaKaRa, Shiga, Japan) with SYBR Premix Ex Taq II (TaKaRa). We used specific primers, PlRep-F3 and PlRep-R3 for PlAMV, AltMV_rt_2280F and AltMV_rt_2425R for AltMV, CymMV-realt6F and CymMV-realt6R for CymMV, LoLV_realt7F and LoLV_realt7R for LoLV, PVM-realt-9F and PVM-realt1R-9R for PVM, TuMV-rt1F and TuMV-rt1R for TuMV, YoMV_rt2F and YoMV_rt2R for YoMV, sGFP-379F and sGFP-486R for *sGFP* mRNA, and actin2F and actin2R for *actin* mRNA. For RT-PCR to detect PlAMV-GFP, we used Pr-det-F and Pr-det-R using cDNA described above as a template.

### Northern blot analysis

Total RNA (1 μg) was analyzed with the digoxigenin (DIG) system (Roche). To produce probes for plus- and minus-strand viral RNA detection, pCR-Pr-1 and pCR-Pr-2 were digested with *Bam*HI restriction enzyme and transcribed with T7 RNA polymerase. The intensities of RNA bands were quantitated using ImageJ software v1.40 (National Institutes of Health).

### Particle bombardment

Particle bombardment was performed using a Biolistic PDS 1000/He Particle Delivery System (Bio-Rad, California, USA), as described previously^56^. The area showing GFP signal was quantitated using ImageJ software v1.40.

### Protoplast preparation and transfection

*Arabidopsis* protoplast preparation and transfection were performed as described previously^57^ with modifications. We added 0.1 M mannitol to W5 solution. For virus inoculation, 100 μg of 35S-driven virus infectious clone was added to 300 μL of protoplast suspension (5 × 10^6^ protoplasts/mL).

### Immunoblotting

The agroinfiltrated leaves and transfected protoplasts were harvested at 4 and 3 dpi, respectively. Total protein extracted using RIPA buffer (50 mmol/L Tris-HCl (pH 8.0), 150 mmol/L NaCl, 0.5 w/v% sodium deoxycholate, 0.1 w/v% SDS, 1.0 w/v% NP-40, 100 mM DTT) with cOmplete^TM^, EDTA-free Protease Inhibitor Cocktail (Roche) was denatured in gel sample buffer (50 mM Tris-HCl (pH 6.8), 2 w/v% SDS, 10% glycerol, 100 mM DTT). Preparation of S30 and P30 fractions was performed as described previously^32^. Protein samples were separated on a 3–8% Tris-Acetate NuPAGE gel (Thermo Fisher Scientific) for RdRp, a 4–12% Bis-Tris gel for TGB1 and CP, and a 12% Bis-Tris gel for TGB2 and TGB3. After electrophoresis, proteins were blotted onto a PVDF membrane and detected with Can Get Signal (TOYOBO, Osaka, Japan). The membrane was stained with Coomassie brilliant blue as loading controls. The intensities of protein bands were quantitated using ImageJ software v1.40 as described previously^56^.

## Additional Information

**How to cite this article:** Keima, T. *et al*. Deficiency of the eIF4E isoform nCBP limits the cell-to-cell movement of a plant virus encoding triple-gene-block proteins in *Arabidopsis thaliana. Sci. Rep.* **7**, 39678; doi: 10.1038/srep39678 (2017).

**Publisher's note:** Springer Nature remains neutral with regard to jurisdictional claims in published maps and institutional affiliations.

## Supplementary Material

Supplementary Information
