# Supplementary material for: Deficiency of the eIF4E isoform nCBP limits the cell-to-cell movement of a plant virus encoding triple-gene-block proteins in Arabidopsis thaliana
Source: Sci Rep. 2017 Jan 6;7:39678. doi: 10.1038/srep39678 (PMC5216350; doi:10.1038/srep39678)
Supplement: Supplementary Information [file srep39678-s1.pdf]

Supplementary information

Deficiency of the eIF4E isoform nCBP limits the cell-to-cell movement of a plant virus encoding triple-gene-block proteins in *Arabidopsis thaliana*

Takuya Keima, Yuka Hagiwara-Komoda, Masayoshi Hashimoto, Yutaro Neriya, Hiroaki Koinuma, Nozomu Iwabuchi, Shuko Nishida, Yasuyuki Yamaji & Shigetou Namba*

a

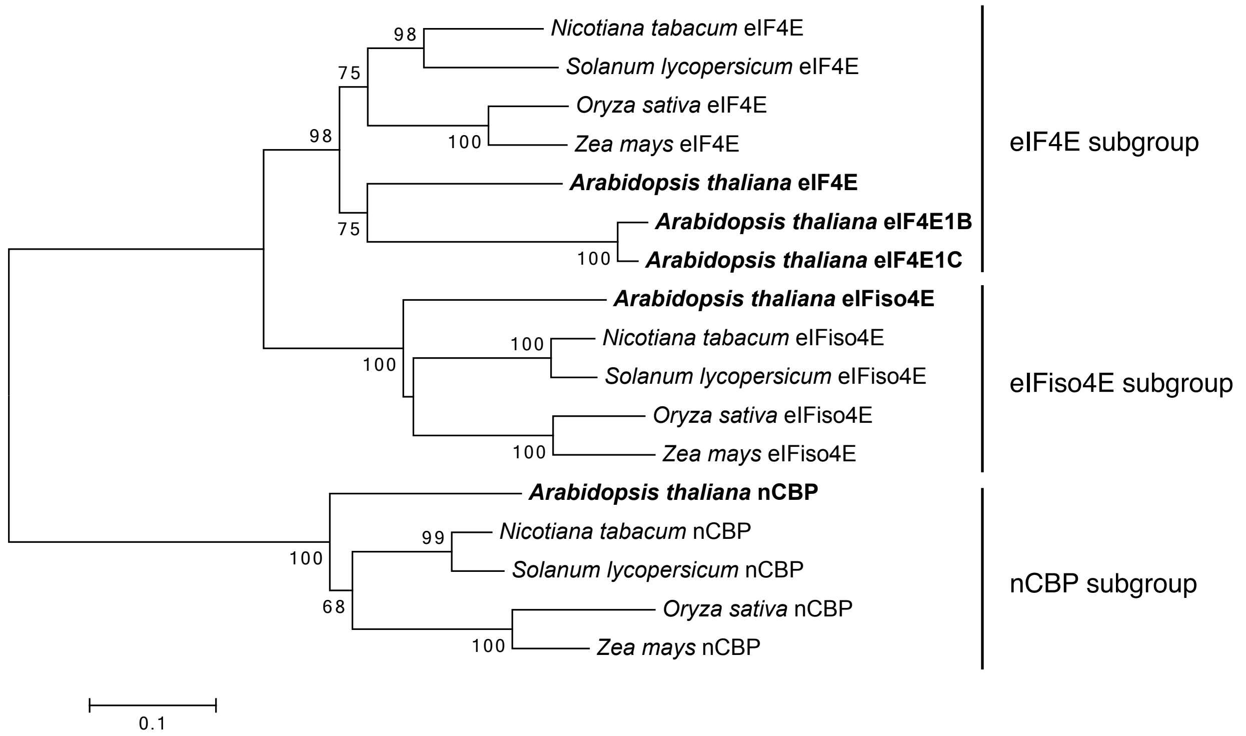
Nicotiana tabacum eIF4E
Solanum lycopersicum eIF4E
Oryza sativa eIF4E
Zea mays eIF4E
***Arabidopsis thaliana* eIF4E**
***Arabidopsis thaliana* eIF4E1B**
***Arabidopsis thaliana* eIF4E1C**
***Arabidopsis thaliana* eIFiso4E**
Nicotiana tabacum eIFiso4E
Solanum lycopersicum eIFiso4E
Oryza sativa eIFiso4E
Zea mays eIFiso4E
***Arabidopsis thaliana* nCBP**
Nicotiana tabacum nCBP
Solanum lycopersicum nCBP
Oryza sativa nCBP
Zea mays nCBP
eIF4E subgroup
eIFiso4E subgroup
nCBP subgroup
98
75
98
100
75
100
100
100
100
99
100
68
100
0.1

b

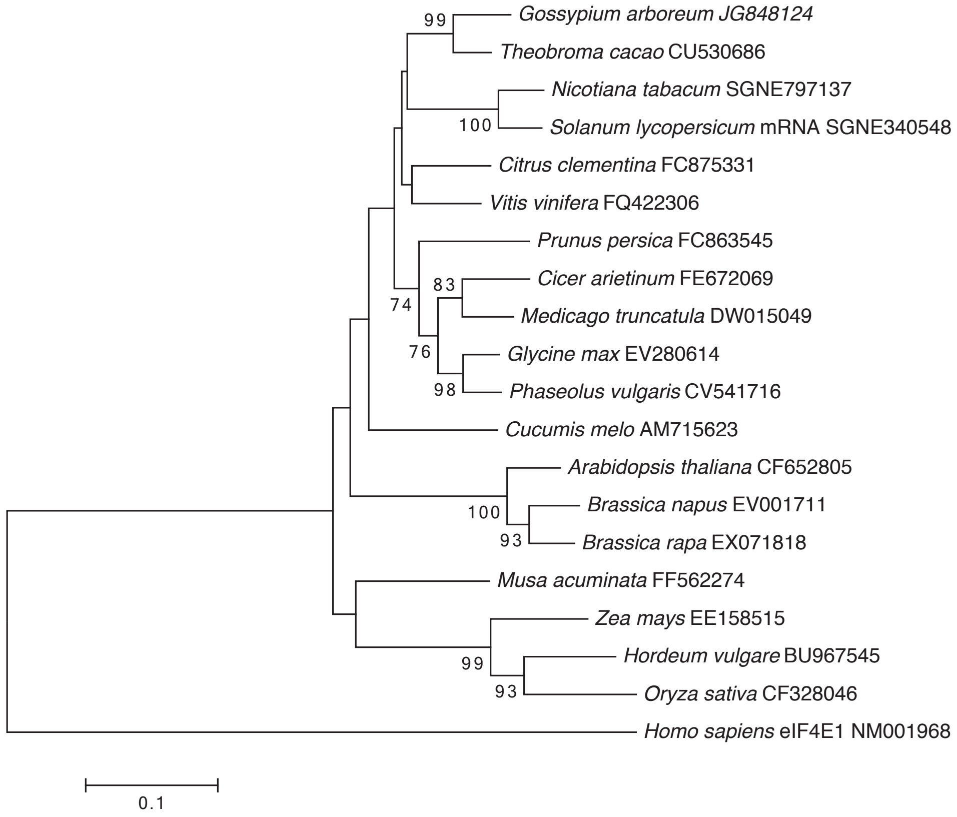
Gossypium arboreum JG848124
Theobroma cacao CU530686
Nicotiana tabacum SGNE797137
Solanum lycopersicum mRNA SGNE340548
Citrus clementina FC875331
Vitis vinifera FQ422306
Prunus persica FC863545
Cicer arietinum FE672069
Medicago truncatula DW015049
Glycine max EV280614
Phaseolus vulgaris CV541716
Cucumis melo AM715623
Arabidopsis thaliana CF652805
Brassica napus EV001711
Brassica rapa EX071818
Musa acuminata FF562274
Zea mays EE158515
Hordeum vulgare BU967545
Oryza sativa CF328046
Homo sapiens eIF4E1 NM001968
99
100
83
74
76
98
100
93
99
93
0.1

c

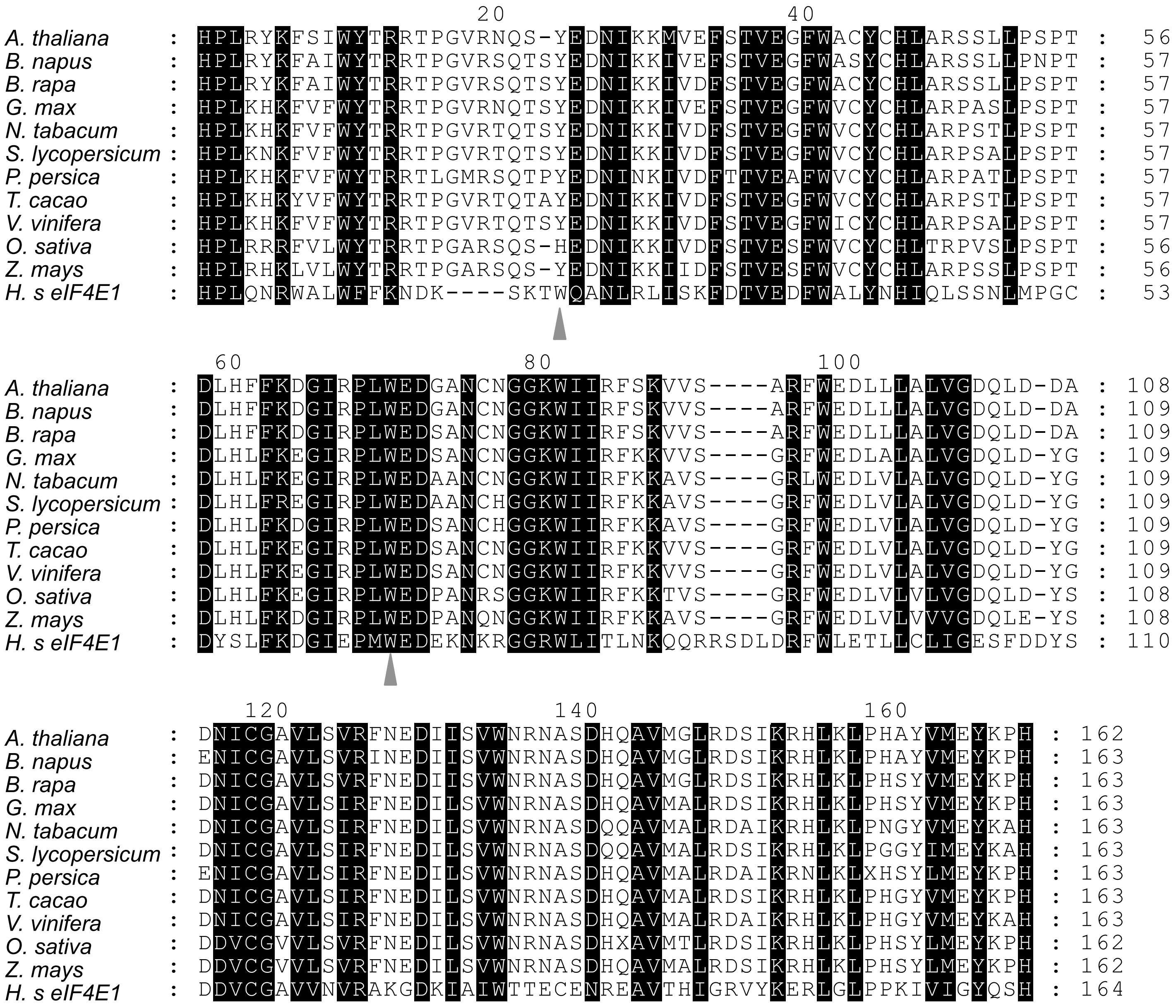

Supplementary Figure S1. Sequence analysis of plant *nCBP* genes

(**a**) Phylogenetic tree of the eIF4E family genes.

(**b**) Phylogenetic tree of the *nCBP* genes from various plant species.

(**c**) Alignment of the amino acid sequences of the core region (from His-43 to His-204 in *A. thaliana* nCBP) of nCBP. Arrowheads indicate conserved Trp residues in eIF4E family proteins[10].

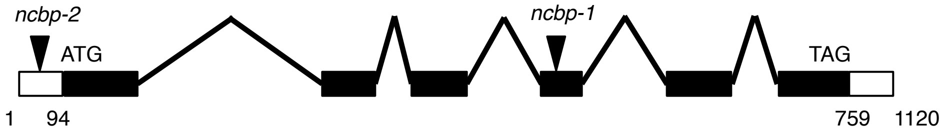

Supplementary Figure S2. Putative structure of the *A. thaliana nCBP* gene
The cDNA structure of the *A. thaliana* nCBP gene. The positions of T-DNA insertions in *ncbp* mutant alleles are indicated.

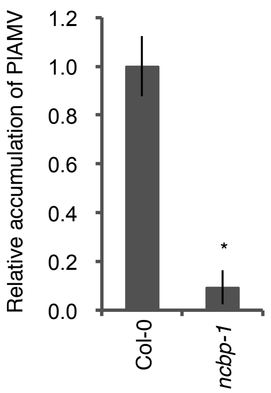

Supplementary Figure S3. Accumulation of PlAMV-GFP RNA in upper leaves of the *ncbp-1* mutant
Total RNA extracted from the upper leaves of the PlAMV-infected *ncbp-1* mutant and Col-0 plants at 4 wpi was subjected to quantitative RT-PCR. Asterisk indicates a significant difference compared with Col-0 (one-tailed Student′s t-test, asterisk; $P < 0.05$).

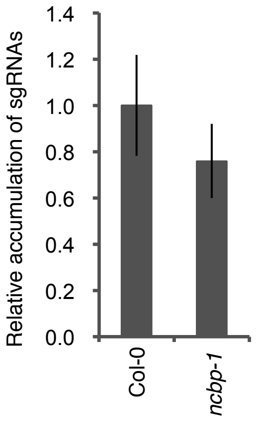

Supplementary Figure S4. Relative accumulation of sgRNAs in virus-infected *ncbp-1* mutant and Col-0 protoplasts

The intensities of the bands for sgRNAs in Figure 4(**b**) were measured and normalized to that for genomic RNA. Accumulation level in Col-0 was used as the standard (1.0). Error bars represent standard errors of three measurements from independent experiments.

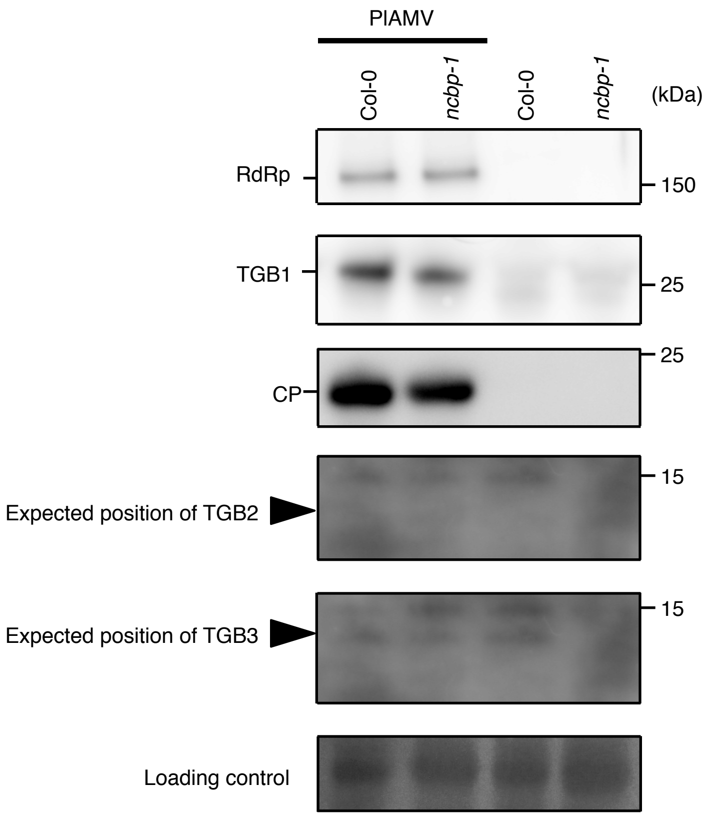

Supplementary Figure S5. Accumulation of viral proteins in the *ncbp-1* mutant and Col-0 protoplasts
Protoplasts prepared from the *ncbp-1* mutant and Col-0 were inoculated with PlAMV. Total protein extracts were subjected to western blot analysis using antibodies against RdRp, TGB1, TGB3, and CP, and antisera against TGB2.

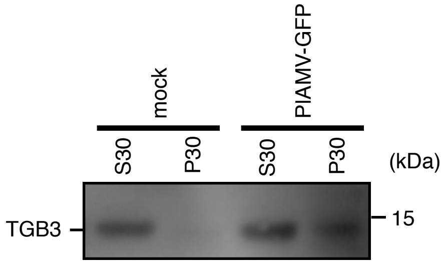

Supplementary Figure S6. Specific detection of TGB3 protein in the insoluble fraction from *A. thaliana* infected by PlAMV

Col-0 was agro-inoculated with PlAMV-GFP. Total protein extracted from inoculated leaves was subjected to ultracentrifugation at 27,000 rpm for 30 min to separate the insoluble (P30) and soluble (S30) fractions. The fractions were analyzed by immunoblotting, using the antibody recognizing TGB3.

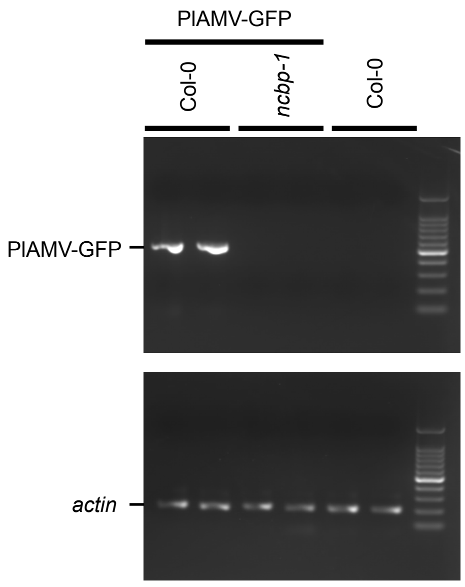

Supplementary Figure S7. Full-length images of RT-PCR analysis in Fig. 1d
See legends of Fig. 1d for detailed information.

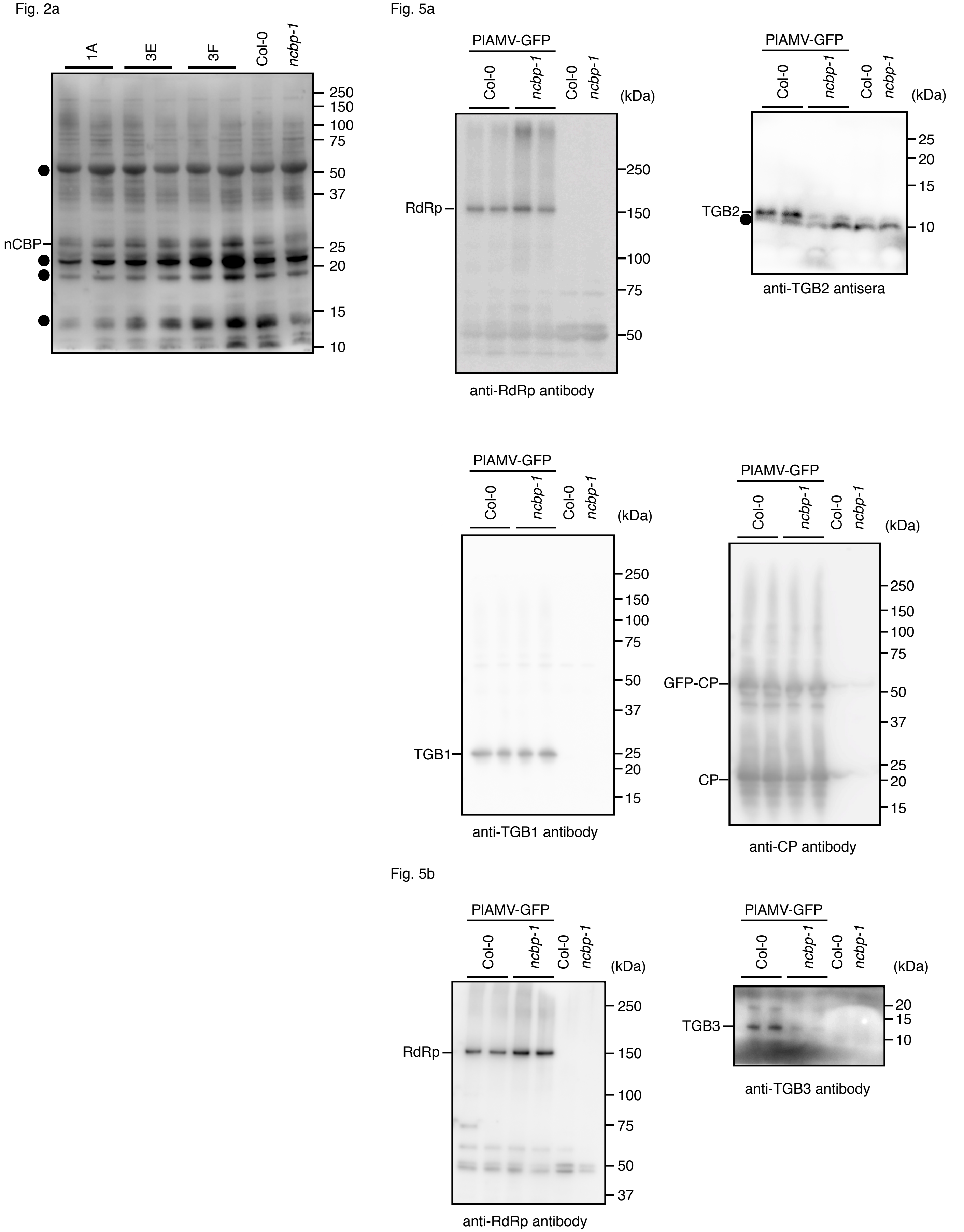

Supplementary Figure S8. Full-length images of western blot analysis

Closed circles indicate nonspecific bands that could be also detected in negative control lanes. See legends of each figure for detailed information.

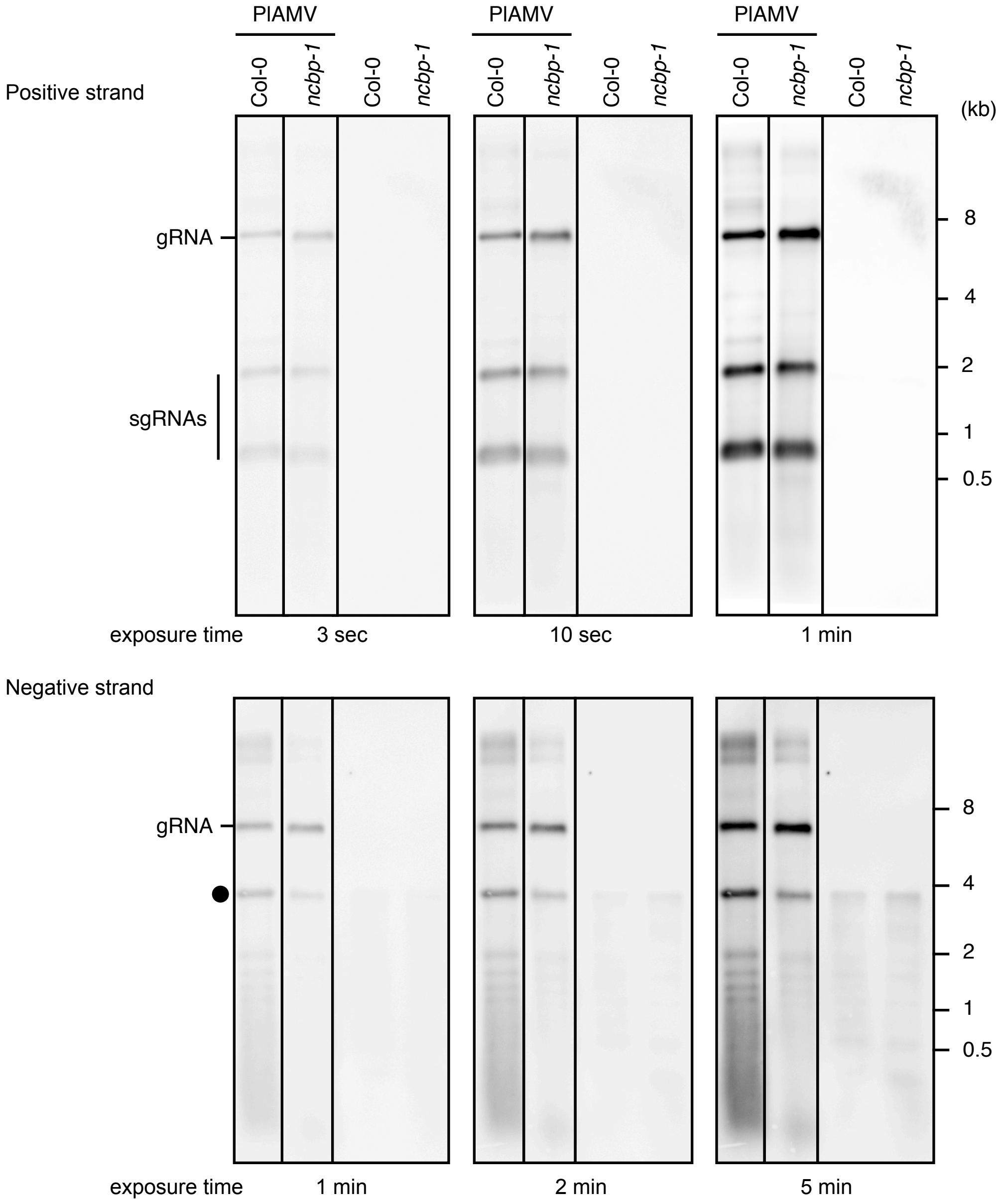

Supplementary Figure S9. Full-length images of northern blot analysis in Fig. 4b with different exposure times

Exposure times are indicated at the bottom of each image. A closed circle indicates a nonspecific band that could be also detected in negative control lanes. See legends of Fig. 4b for detailed information.

Supplementary Table S1. The efficiency of PlAMV systemic infection in the *ncbp* mutants and complemented lines

	3 wpi			4 wpi		
	Detected[a]	Total	Ratio (%)	Detected[a]	Total	Ratio (%)
ncbp-1	0	4	0	3	5	60
ncbp-2	0	6	0	0	6	0
Col-0	4	4	100	3	3	100
#1A	5	5	100	NT	NT	NT
#3E	5	5	100	NT	NT	NT
#3F	5	5	100	NT	NT	NT

[a]Plants were mechanically inoculated with PlAMV-GFP and virus was detected by RT-PCR at 3 week-post inoculation (wpi) or 4 wpi. The numbers of systemically infected plants were shown. NT: not tested.

Supplementary Table S2. Primers used in this study

Name	Sequence (5'-3')
PlRep-F3	AATCCCCAGACTTCCATGAGCACC
PlRep-R3	TTTTCTTTGCGCCGAGCTTCTC
actin2F	GCACCCTGTTCTTCTTACCG
actin2R	AACCCTCGTAGATTGGCACA
Pr-det-F	AACTGGCCACAATCACCCAGGGG
Pr-det-R	CTAGTCGGAGGGGGAAGGG
AltMV_rt_2280F	CCCCACTCCCTTTTCTCC
AltMV_rt_2425R	ATTGGCGTTGACCATTCTCC
CymMV-realt6F	CCCCGAGGATGTTATAGAAGGA
CymMV-realt6R	GGTATCTGGTGGCGTTGTAGG
LoLV_realt7F	CAGCAATGCGAGGGACTATCTAC
LoLV_realt7R	TGTCGGGGTTTGAGTTTGG
PVM-realt9F	GCTACAGGTGTGCTTGGATCTG
PVM-realt1R-9R	TGCCCGACCCAAAAGTG
TuMV-rt1F	GGAGGAGGAGAAGAAGGAGAGAG
TuMV-rt1R	TCCAGAGGTTCCAGCGTTTAC
YoMV_rt2F	TACTACAACGCTTTATCCGAGCTGTC
YoMV_rt2R	TTATCGCCACCACCACCTTTG
sGFP-379F	AAGGGCATCGACTTCAAGGAG
sGFP-486R	GATGCCGTTCTTCTGCTTGTC
Sl-At5g18110-up1374F	ACGCGTCGACATTAGTTATACACTTTTCC
Nt-At5g18110-down1011R	ATAAGAATGCGGCCGCTGTTGCCAAAGAATTGG
